# Vigilance behaviour during the calving season in female Tibetan antelopes (*Pantholopshodgsonii*)

**DOI:** 10.3897/BDJ.11.e107957

**Published:** 2023-09-05

**Authors:** Peiwei Li, Hongfeng Zhang, Dongdong Yang, Congran Gong, Dong Wu, Yuting Sun, Yan Liu, Junqing Tang, Han Hu, Qiupei Zhaxi, Wei Xu, Lina Su, Yinhu Li, Xiaomin Wu

**Affiliations:** 1 Shaanxi Institute of Zoology, xi'an, China Shaanxi Institute of Zoology xi'an China; 2 School of Urban Planning and Design, Shenzhen Graduate School, Peking University, Shenzhen, China School of Urban Planning and Design, Shenzhen Graduate School, Peking University Shenzhen China; 3 Hoh Xil Nature Reserve service, Golmud, China Hoh Xil Nature Reserve service Golmud China; 4 Three-River-Source National Park Service, Xining, China Three-River-Source National Park Service Xining China

**Keywords:** Tibetan antelope, calving, vigilance, Zonag Lake

## Abstract

Tibetan antelopes (*Pantholopshodgsonii*) migrate great distances to specific delivery and calving areas. In the current study, we investigated calving site selection and vigilance behaviour during delivery and nursing in migratory female Tibetan antelopes at Zonag Lake. According to observations and analysis, the females were distributed south of Zonag Lake, where vegetation was abundant. We determined their dates of migration (crossing the Qinghai-Tibet Highway observation site), showing a shift of one month during the period from June in 2008 to May 2021. Results also showed that 81.4% of females expressed high vigilance behaviour during calving and nursing compared to those without calves (7.1%). From delivery until calf standing, females were highly vigilant and spent considerable time scanning, with 96% of females showing vigilance behaviour. Females with calves (average 9.94 ± 0.62 s) spent more time on vigilance behaviour than females without calves (average 6.25 ± 1.38 s). Females with newborns spent the greatest amount of time being vigilant (average 51.63 ± 4.24 s). These results not only identify basic Tibetan antelope calving behaviour, but also provide scientific analysis and evidence for further ethological research on female Tibetan antelopes.

## Introduction

The Tibetan antelope (*Pantholopshodgsonii*), also known as chiru, is a flagship species endemic to the Qinghai-Tibet Plateau. It is distributed in plateau areas above 4 000 m altitude, including Qiangtang in Tibet, Altun in Xinjiang and Hoh Xil and Sanjiangyuan in Qinghai (Li and Jiang 2008, Luo et al. 2018). Historical hunting and habitat loss greatly limited antelope migration, leading to dramatic population declines (Zhang et al. 2013). The species was designated as Near Threatened by the IUCN in 2021 and classified as Category I (Endangered in China) by the National Protected Wild Animal Species in China in 1989 until now. Although the Tibetan antelope population demonstrates an increase in recent years due to reduced hunting pressure, habitat fragmentation and pasture-land development continue to have an impact on population expansion (Bai et al. 2018, Chen et al. 2019). Therefore, investigating the behaviour of Tibetan antelopes could help provide insight into the environmental adaptability and regulatory mechanisms of plateau animals and provide basic knowledge for Tibetan antelope conservation (Ge et al. 2013, Signore and Storz 2020). Due to the high altitude and harsh conditions of the Qinghai-Tibet Plateau, many species exhibit hypoxia adaptation or oxygen sensing. In large-sized high-altitude animals, high rates of metabolism may induce greater transformation to oxygen homeostasis and complex oxygen delivery systems, such as the lungs, heart and vasculature, have evolved to require precise dynamic control (Chang Rong and Zhen-zhong 2012, Tong et al. 2013, [Bibr B9883729][Bibr B9883738]).

Migration is a strategy employed by a broad range of taxa in response to temporally and spatially changing environmental conditions. Multiple factors can drive animal migration, which usually occurs regularly (Couzin 2018). In some species, the desire for migration is caused by homing (Andreychev and Kiyaykina 2020). Different animals have a specific reason for migrating. In Tibetan antelopes, thousands of females migrate to find suitable areas for delivery and nursing and entire populations divide to form resident and migratory subpopulations (Festa Bianchet et al. 2019). Before giving birth, most female Tibetan antelopes migrate along a set route to the Sun Lake, Zonag Lake, in Hoh Xil, Qinghai, Tianshui River in Qiangtang, Tibet and Muzitag Peak in Altun, northwest of the Qinghai-Tibet Plateau (Xiaomin and Hongfeng 2011). They return to their original habitat after giving birth. Thus, Tibetan antelope migration is primarily caused by maternal behaviour, i.e. calving. In contrast, other species, such as wildebeest (*Connochaetes Burchell*) and caribou (*Rangifertarandus* caribou Gmelin), migrate together as families in search of better climate and vegetation (Bauer et al. 2009, Bowlin et al. 2010). Several studies have hypothesised that animal migration follows seasonal changes in environmental quality, with migratory animals having greater access to nutritious food than sedentary animals (Seidler et al. 2015). Large artiodactyl groups make it more difficult for predators to hunt. These migrations have site fidelity, which may also be conditional on the success of animals’ recent visits to that location and it may become stronger with age as the animal gain experience in their landscape (Morrison et al. 2021). In the current study, we investigated the calving locations of female Tibetan antelopes and analysed their vigilance behaviour during nursing around Zonag Lake (Sanjiangyuan National Nature Reserve). As the eastward and westward herds of female Tibetan antelopes follow the classic migration route across the Qinghai-Tibet Highway (QTH), QTH is the site of female Tibetan antelopes’ migration and is close to the Zonag Lake. We established observation sites at the crossing points (35°19'N, 93°35'E) (Festa Bianchet et al. 2019). However, the seasonal migration in female Tibetan antelopes is still unknown. Research on Tibetan antelopes remains limited, particularly long-term studies on behaviour and physical regulatory mechanisms.

In this study, we focus on the vigilance behaviour of female Tibetan antelopes during their calving season, which has not been studied before. Thousands of female Tibetan antelopes leave their males and starts their migration for calving every year. Under this situation, female Tibetan antelopes need to pay more attention to their predator with high vigilance. Our research is aimed to obtain detailed scientific data regarding female Tibetan antelope vigilance behaviour during calving. This study should provide evidence and methods for further research on Tibetan antelope behaviour and a theoretical foundation for population and habitat protection.

## Materials and Methods

### Study area

Research was conducted around Zonag Lake (35°28’–35°37’N, 91°46’–92°06’E) in Hoh Xil, Qinghai Province, China. Average altitude is about 4 400 m, with an elevational drop of 300 m from west to east. The lowest recorded temperature is −41℃, historically. The climate is cold, with strong winds, high levels of solar radiation and higher precipitation in summer. Dominant vegetation in this alpine desertificated steppe ecosystem includes *Stipapurpurea* and *Carexmoorcroftii*. According to our investigation, we found that Tibetan antelope distributed in stipa steppe areas.

### Calving behaviour observations and sampling

Behavioural observations of the female Tibetan antelopes were performed using binoculars (8X42, BOSMA, Guangzhou, China) and videos were taken at a distance 200m to avoid observer influence. Observations and recordings were conducted from 10:00 am to 6:00 pm (Female tibetan antelopes' activity and feeding time) and continued for the entire calving season.

**Figure 1.**
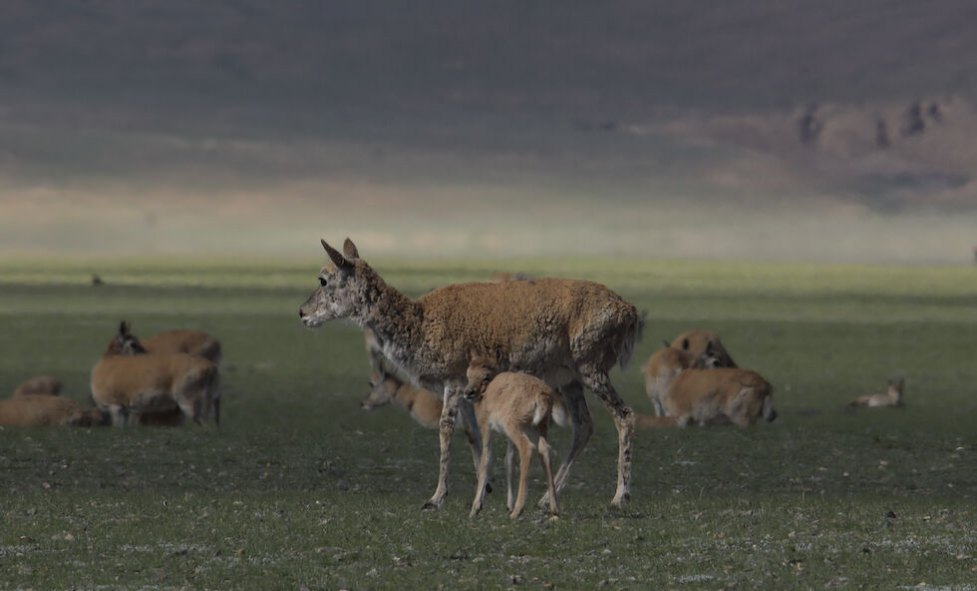
Female Tibetan antelope exhibiting vigilance behaviour.

Observations were conducted at Zonag Lake from May to August 2019. We observed 332 females in total, accounting for 2000 min of observation time. Newborn calves are calves which could not stand after initial delivery. Vigilance behaviour of the female Tibetan antelopes was observed and recorded after calves were delivered and first nursed. (1) Vigilance behaviour was defined as heads raised or scanning, with the angle between the neck and foreleg always significantly greater than 120° (Fig. 1). Vigilance suggests that all sensory organs are alert to potential predators. (2) Vigilance level was defined as the duration and percentage of individuals scanning and vigilance frequency was defined as the number of scans per individual in 600 s. Moving was defined as walking or running with their head at or above the shoulder level. (3) Other behaviour: resting was recorded if the animal was lying down. These data were compared to female Tibetan antelopes without calves. Only one to four individuals were observed in each group in order to reduce the probability of group size's and groups at a particular location were revisited on subsequent days only if they contained more than 10 individuals.

### Camera trapping

Observation sites suitable for female Tibetan antelopes were selected around QTH. Each camera (Ltl-6210MC•Plus, Ltl Acorn Electronics Co., Ltd., Shenzhen, China) site covered 20 km. Each observation site was divided into 1 × 1-km grids (20 grids for each sample site). The distance between two camera traps in the different grids was at least 500 m. The camera sensor was parallel to the ground to avoid direct sunlight. Bio-Photo v.2.1 was used to obtain video information and the whole recording times was more than 30,000 hours.

### Unmanned aerial vehicle (UAV) remote sensing

The UAV consisted of a drone flight platform and a GPS navigator with various sensors. The UAV acquired high-resolution remote sensing images of Tibetan antelopes to determine their distribution around Lake Zonag. UAV used in our study was made by Northwestern Polytechnical University, for animal monitoring, flying up to 700 m high above the ground with low noise. Research showed that the population number of Tibetan antelopes in area (0 ~ 100, 100 ~ 200 m) near the UAV lane did not show a significant difference of colour between Tibetan antelopes and land. The images were used to analyse body colour and identify animals with Pix4Dmapper. The Tibetan antelopes were recognised, based on their tawny colour with distinct shades of black and the shape length is between 20 and 35 pixels. The images were segmented using the threshold obtained by the minimum automatic threshold algorithm and then morphological filtering and contour detection were performed to determine the Tibetan antelope distribution.

### Moderate-resolution Imaging Spectroradiometer - NDVI (Normalised Difference Vegetation Index)

The NDVI is a good indicator of vegetation growth and is closely related to leaf area, biomass and vegetation coverage (Matese and Gennaro 2021). Female Tibetan antelopes come to Zonag Lake every year to calve, the possible reason might be sufficient food resource around the Lake. For this reason, we chose the NDVI map of Zonag Lake to test the distribution of female Tibetan antelopes. The NDVI dataset was obtained from Hoh Xil Nature Reserve service (accessed 15 days on July 2019 (3 July to 17 July)) and has been verified by field accuracy verification. NDVI values were calculated by the band Math of Environment for Visualizing Images (ENVI) software: NDVI =(R_nir_-R_red_)/ (R_nir_+R_red_). NDVI is also a sensitive index to reflect the dynamic changes of regional vegetation. After data format unification, coordinate conversion and weather interference elimination using the maximum synthesis method, we obtained the NDVI to determine vegetation species richness around Zonag Lake during the calving season. NDVI > 0.45 represents areas of medium vegetation cover, 0.15 < NDVI < 0.3 represents areas of low vegetation cover, NDVI < 0.15 represents areas of very low vegetation cover (Classification standard of NDVI, Ministry of Water Resource of China, 2008) (Huang et al. 2019).

## Data resources

Vigilance level and frequency were determined for 332 Tibetan antelopes with calves. Data were tested for normality (*P* < 0.001) with the one-sample Kolmogorov-Smirnov test. Vigilance duration and frequency showed normal distribution.

Vigilance duration and frequency of all female Tibetan antelopes were analysed by two-tailed Student’s *t-Test* using SPSS v.22.0. Vigilance duration was shown as mean ± SEM. All significant differences were set at *P* = 0.05, with * and ** indicating significant difference at *P* < 0.05 and *P* < 0.01, respectively and "ns" indicating not significant.

## Results

### Arrival time of female Tibetan antelopes and distribution of Tibetan antelopes around Zonag Lake

**Figure 2.**
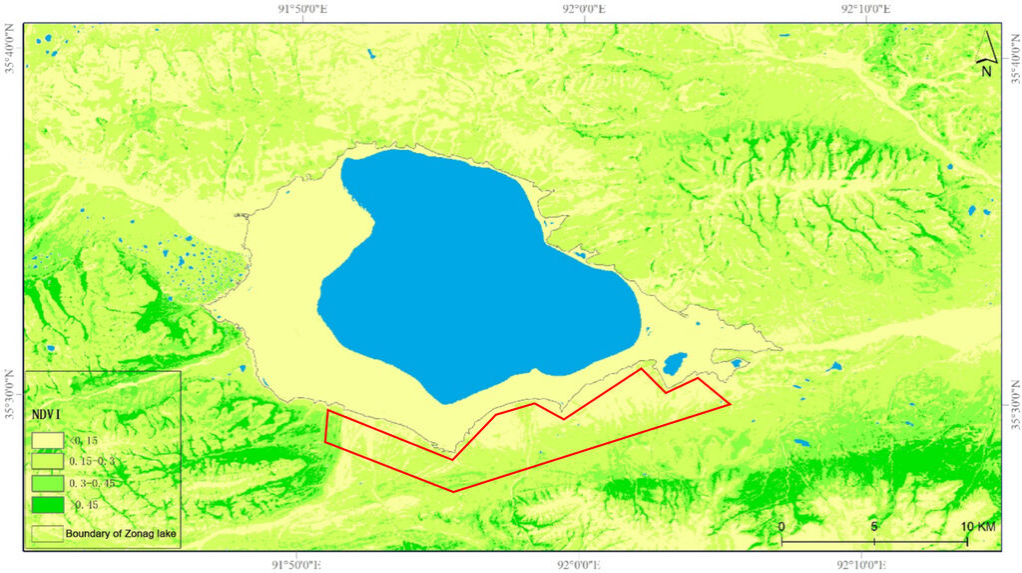
Distribution of female Tibetan antelopes around Zonag Lake in July 2019 (red zone indicates main distribution area of female antelopes).

Based on the monitoring data of the QTH observation sites from 2008 to 2021, we determined the dates when the female Tibetan antelopes first crossed the QTH to reach the calving area, as shown in Table 1. These records showed that migration and arrival times have advanced nearly one month since 2015, with arrival occurring 34 days earlier in 2020 than in 2008. This trend suggest that antelopes are arriving at Zonag Lake and preparing for delivery earlier than in previous years. The UVA images and results showed that female Tibetan antelopes were distributed south of Zonag Lake in 2019 (Fig. 2).

### Vigilance behaviour of female Tibetan antelopes during nursing

**Figure 3.**
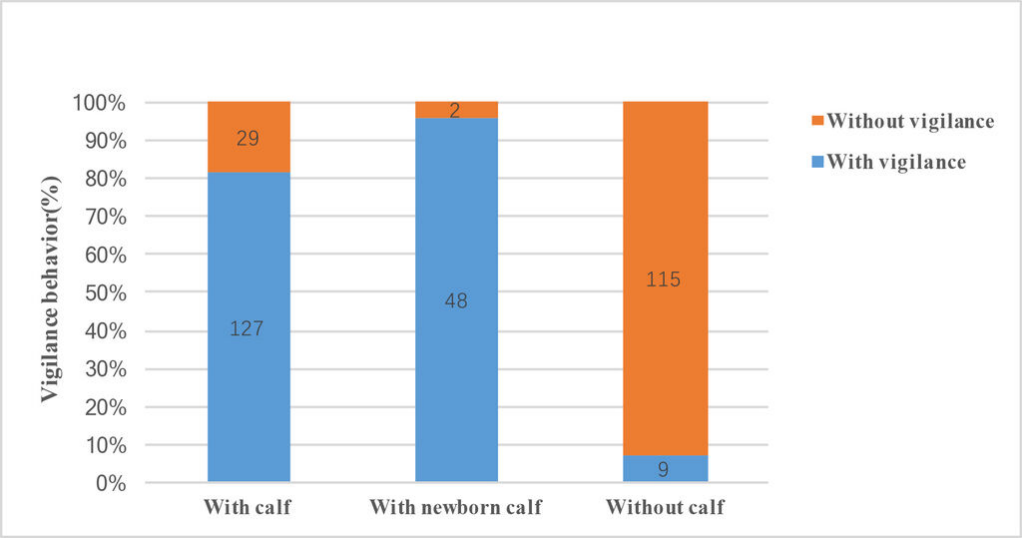
Effect of nursing status on vigilance behaviour in female Tibetan antelopes.

**Figure 4.**
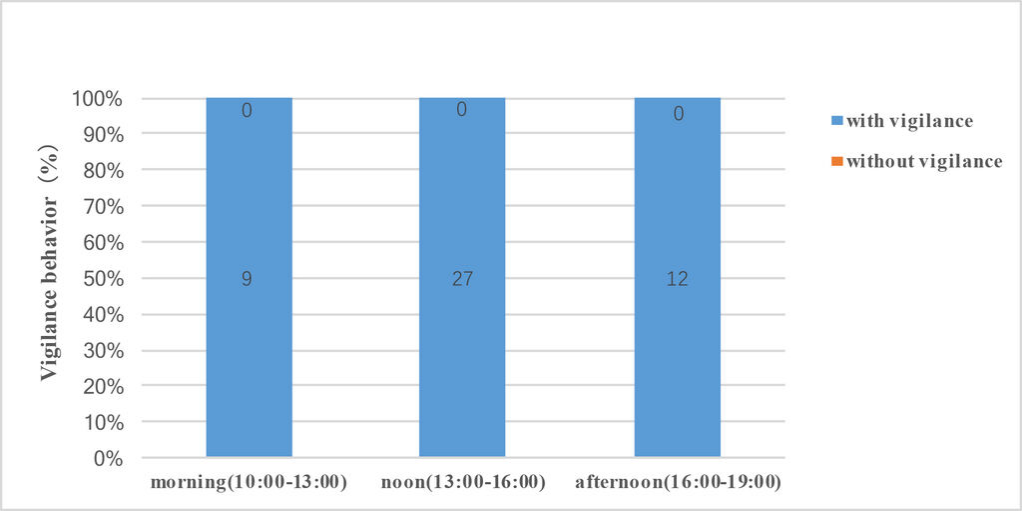
Effect of nursing status on vigilance behaviour in female Tibetan antelopes.

We found that the female Tibetan antelopes exhibited increased vigilance behaviour when they had a calf to nurse. Data on alert posture duration are shown in Fig. 3. Notably, 81.4% (127/156) of females with calves displayed vigilance behaviour and 96% (48/50, two newborn calves died immediately and their mothers left) of females with newborns showed vigilance behaviour compared to 7.1% (9/126) of females without calves. Based on the 48 female Tibetan antelopes with newborns, we found that the vigilance behaviour was not affected by time, they continued to express vigilant behaviour well after giving birth (Fig. 4).

**Figure 5.**
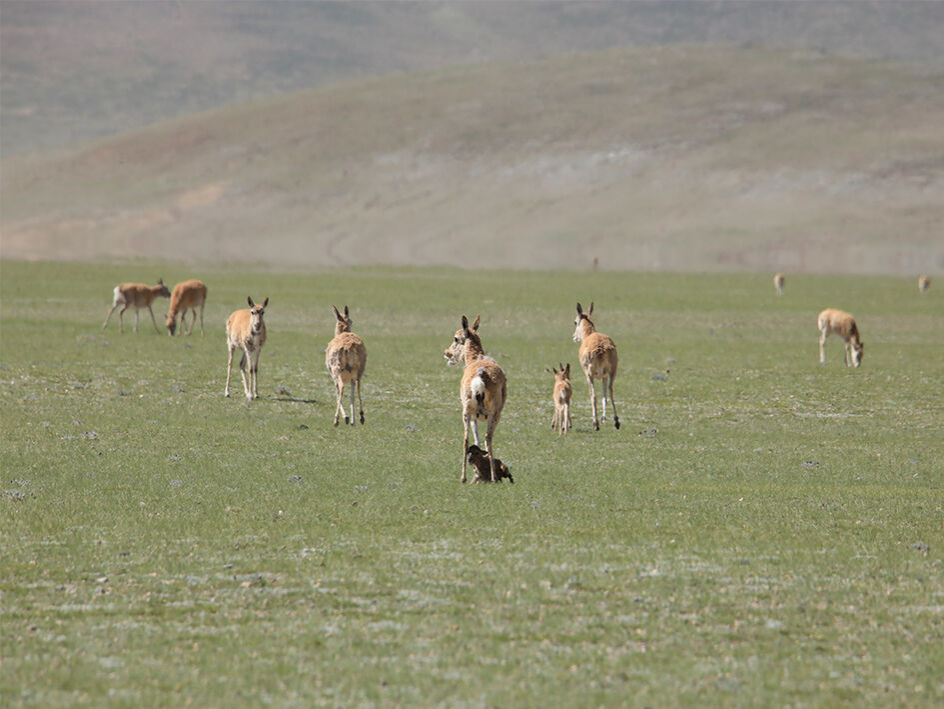
Female Tibetan antelope with newborn calf exhibiting vigilance behaviour.

Furthermore, females displayed high vigilance and prolonged scanning immediately after delivery until the calf could stand (Fig. 5). After delivery, females preferred to keep their newborn under their belly, licking and cleaning the calves for about 20 min.

**Figure 6.**
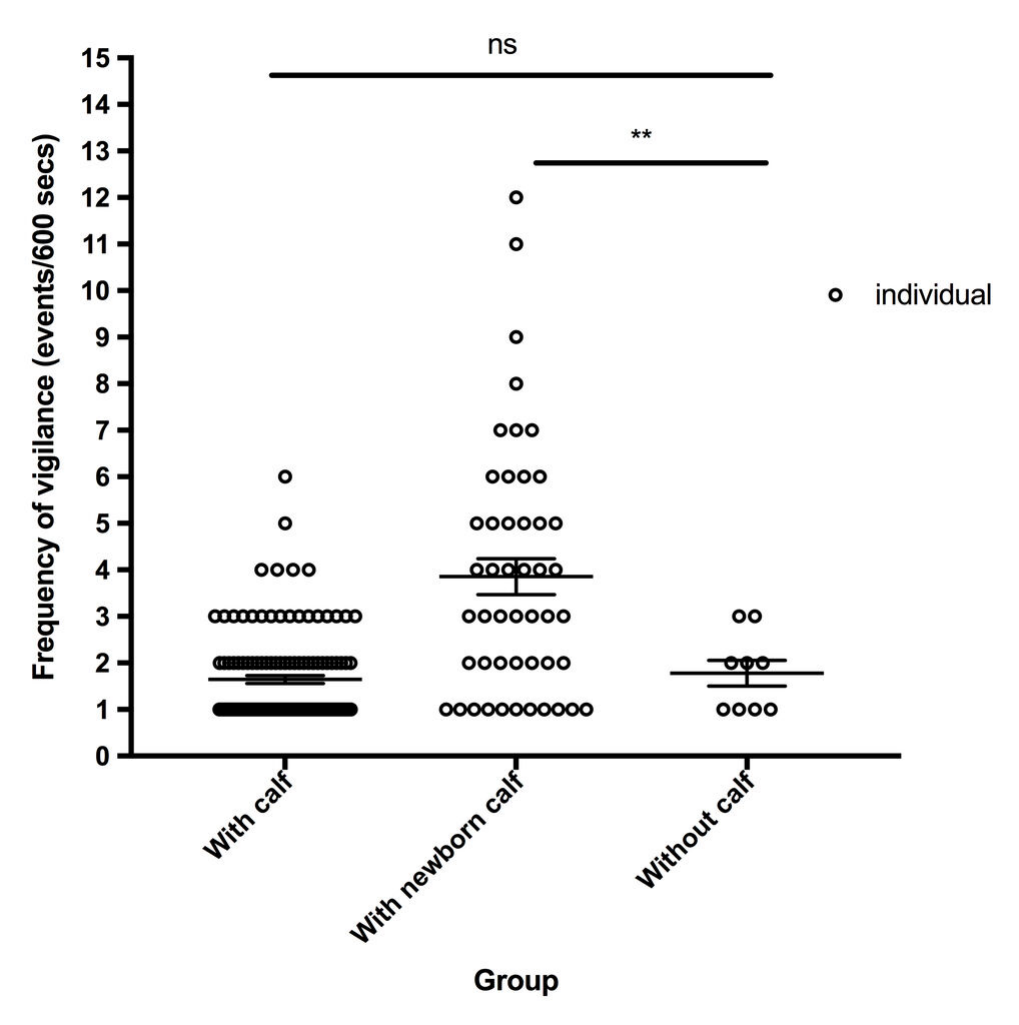
Fig 6. Frequency of vigilance in female Tibetan antelopes.

Based on vigilance time (Suppl. material 1), females with calves (2–53 s, average 9.94 ± 0.62 s, median 6 s) spent more time on vigilance behaviour than females without calves (2–21 s, average 6.25 ± 1.38 s, median 3.5 s). Females with newborns spent the greatest amount of time being vigilant (3–322 s, average 51.63 ± 4.24 s, median 31.3 s). As shown in Fig. 6, amongst the three groups, vigilance frequency was also highest for females with newborns.

## Discussion

Previous research suggests that female Tibetan antelopes typically start their migration in June, which lasts for about 2 months over the summer season (Lian et al. 2007a, Rivrud et al. 2019). In our study, however, during the last decades, the migration period appears to be shifted to earlier seasons, from early June in 2008 to early May in 2021. The may be related to climate change and its association with increasing environmental variability (Dongdong et al. 2017). Environmental changes can influence the relative benefits of strong or weak site fidelity by altering forage abundance, community composition and distribution (Fleming et al. 2016, Abrahms et al. 2018). The period of migration and arrival at Zonag Lake may be related to the ungulates’ study and dissemination of seasonal distribution of food. Our findings suggest that vegetation may recover in the southern areas of Zonag Lake earlier, thus providing the migratory females with sufficient food for delivery, with the evidence of NDVI data (Haidi et al. 2014).

Previous studies have found that ungulate migration is a strategy for exploiting altitudinal, longitudinal and other topographic gradients of plant phenology that determine forage quality (Abrahms et al. 2018). Animals gain information on sources of high-quality vegetation/food, for example, *Oviscanadensis* and *Alcesalces* migrate to new green habitats with historical populations that have lasted for hundreds of years; this trend would influence the individuals which did not migrate initially(Randler 2005, Sasaki and Biro 2017). This social learning would increase their propensity to migrate. Some species migrate annually in mixed groups (males and females together) for sufficient food and better places, such as wildebeest (*Budorcastaxicolor*) and pronghorns (*Antilocapraamericana*). However, for Tibetan antelopes, males do not migrate, only female Tibetan antelopes gather and migrate in order to find calving places with good conditions and to nurse calves. Our observations support this view, as female Tibetan antelopes migrated annually to Zonag Lake to obtain sufficient food and water for birthing and nursing.

This research on female Tibetan antelopes’ vigilance behaviour during nursery reminded us that it is only female Tibetan antelope which group together for the annual migration to deliver and nurse calve when we compared these data to other ungulates, such as female Tibetan gazelles (*Procaprapicticaudata*) (Randler 2005). A previous study also showed that their vigilance duration was shorter than female Tibetan antelopes in same group size (Li and Jiang 2008). Our study suggested the female Tibetan antelopes with calves, especially newborns, were more vigilant. The reason for this may be that, in mixed groups, male Tibetan antelopes take more responsibility for vigilance. Only female Tibetan antelopes delivered and nursed alone before they migrated and returned to the habitat (Xiaomin and Hongfeng 2011). Therefore, females must assume vigilance behaviour during birthing and nursing until they return to the main group. In addition, we also found that female Tibetan antelopes which had newborn calves were in high and constant vigilance before the calve could stand or move. Besides, female Tibetan antelopes remained close to their calves. In giraffes (*Giraffacamelopardalis*), females with young calves are more frequently found close to traditional homesteads (bomas) compared to females without young calves (Scheijen et al. 2021). In addition, while male impalas (*Aepycerosmelampus*) show greater vigilance than females (Olivier Pays et al. 2012), females in other species show a higher level of group vigilance than males (Michelena et al. 2006, Barnier et al. 2016). Additionally, females present greater vulnerability to predators when nursing calves. Previous research has also found that vigilance time and frequency decrease with increased group size in Tibetan antelopes due to the decrease in the risk of predation, unlike traditional predators of Tibetan antelopes, like bears (*Ursusarctos*) and wolves (*Canislupus*) (Lian et al. 2007b) (Suppl. material 2) (Suppl. material 3). We also found ravens (*Corvuscorax*)(Suppl. material 4) directly attack the eyes and stomach region of calves. Under these circumstances, female antelopes with newborns must be on high alert and provide protection for their calf, as evidenced in our study. We also found that after the calves grew and acquired the ability to move and forage independently, the length and frequency of female vigilance behaviour showed a decreasing trend.

## Conclusions

Our results demonstrated that female Tibetan antelopes preferred areas on the southern bank of Zonag Lake for feeding and delivering calves. Amongst female Tibetan antelopes, those with newborns showed the highest vigilance. A future study will focus on the potential involvement of hormones in vigilance behaviour of female Tibetan antelopes as well as the underlying regulatory mechanism.

## Supplementary Material

2361B8A9-215D-5D6C-99BD-A0AD1618D96B 10.3897/BDJ.11.e107957.suppl1 Supplementary material 1 Data of vigilance Data type Excel File: oo_860573.xlsx https://binary.pensoft.net/file/860573 Peiwei Li

B7924FB9-9664-5FBD-877C-6EA5C9649554 10.3897/BDJ.11.e107957.suppl2 8324744 Supplementary material 2 Predators: wolf Data type images File: oo_896131.png https://binary.pensoft.net/file/896131 Peiwei Li

E8D44583-C643-5B93-9D3E-95422C06A936 10.3897/BDJ.11.e107957.suppl3 Supplementary material 3 Predators: bear Data type image File: oo_896132.JPG https://binary.pensoft.net/file/896132 Peiwei Li

622AF94E-1D0C-5142-A74E-6E7BF7A15E6C 10.3897/BDJ.11.e107957.suppl4 Supplementary material 4 Predators: *Corvuscorax* Data type image File: oo_896134.JPG https://binary.pensoft.net/file/896134 Peiwei Li

## Figures and Tables

**Table 1.** Dates when female Tibetan antelopes crossed QTH from 2008 to 2021.

Year	Date	Year	Date
2008	3 June	2009	21 May
2010	12 May	2011	11 May
2012	17 May	2013	10 May
2014	12 May	2015	1 May
2016	8 May	2017	13 May
2018	3 May	2019	15 May
2020	30 April	2021	2 May
