# Supplementary figures and images for: Vigilance behaviour during the calving season in female Tibetan antelopes (Pantholopshodgsonii)
Source: Biodivers Data J. 2023 Sep 5;11:e107957. doi: 10.3897/BDJ.11.e107957 (PMC10498271; doi:10.3897/BDJ.11.e107957)

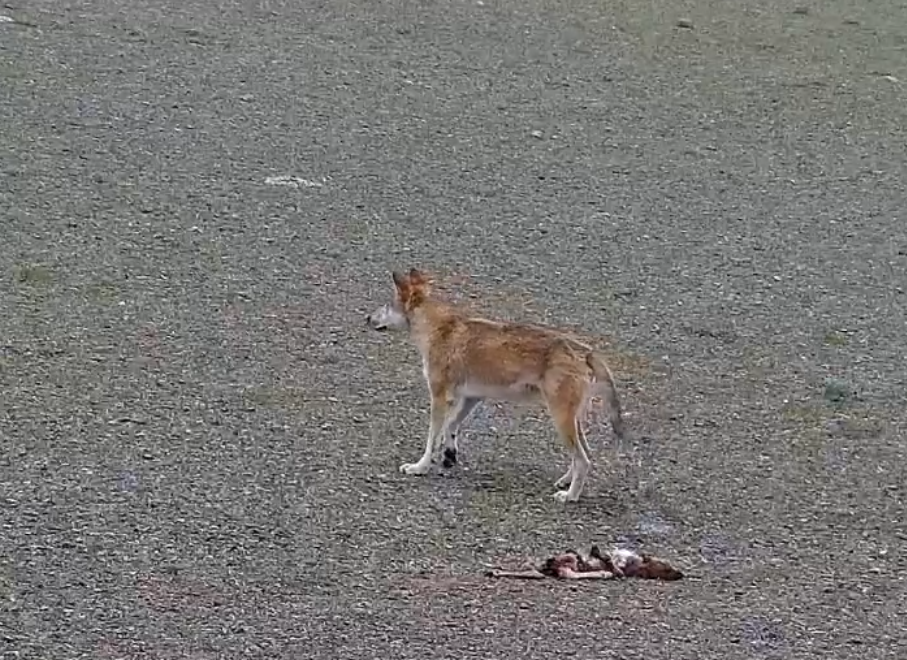

Supplement: Supplementary material 2 — Predators: wolf [file bdj-11-e107957-s002.png]

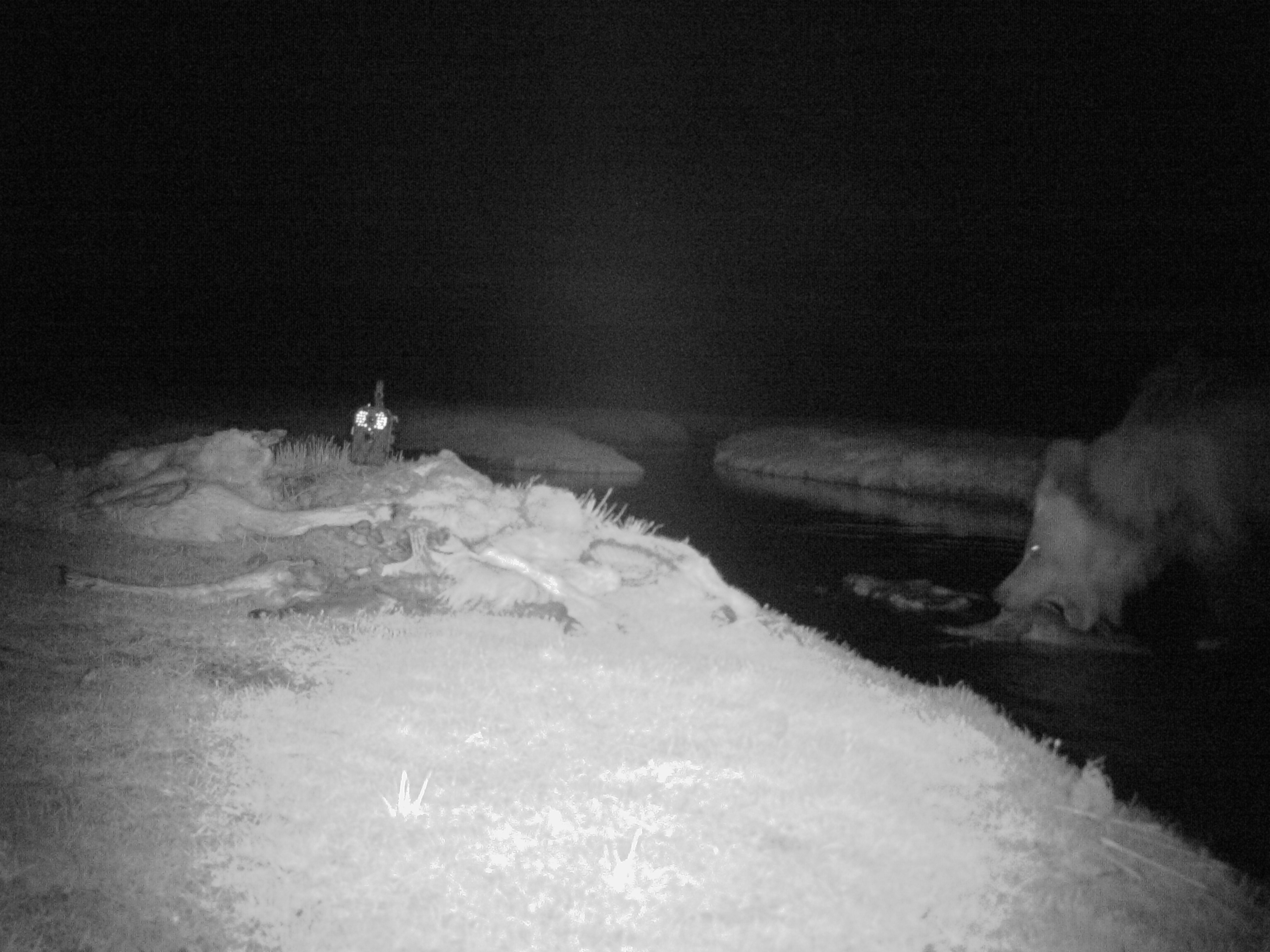

Supplement: Supplementary material 3 — Predators: bear [file bdj-11-e107957-s003.jpg]

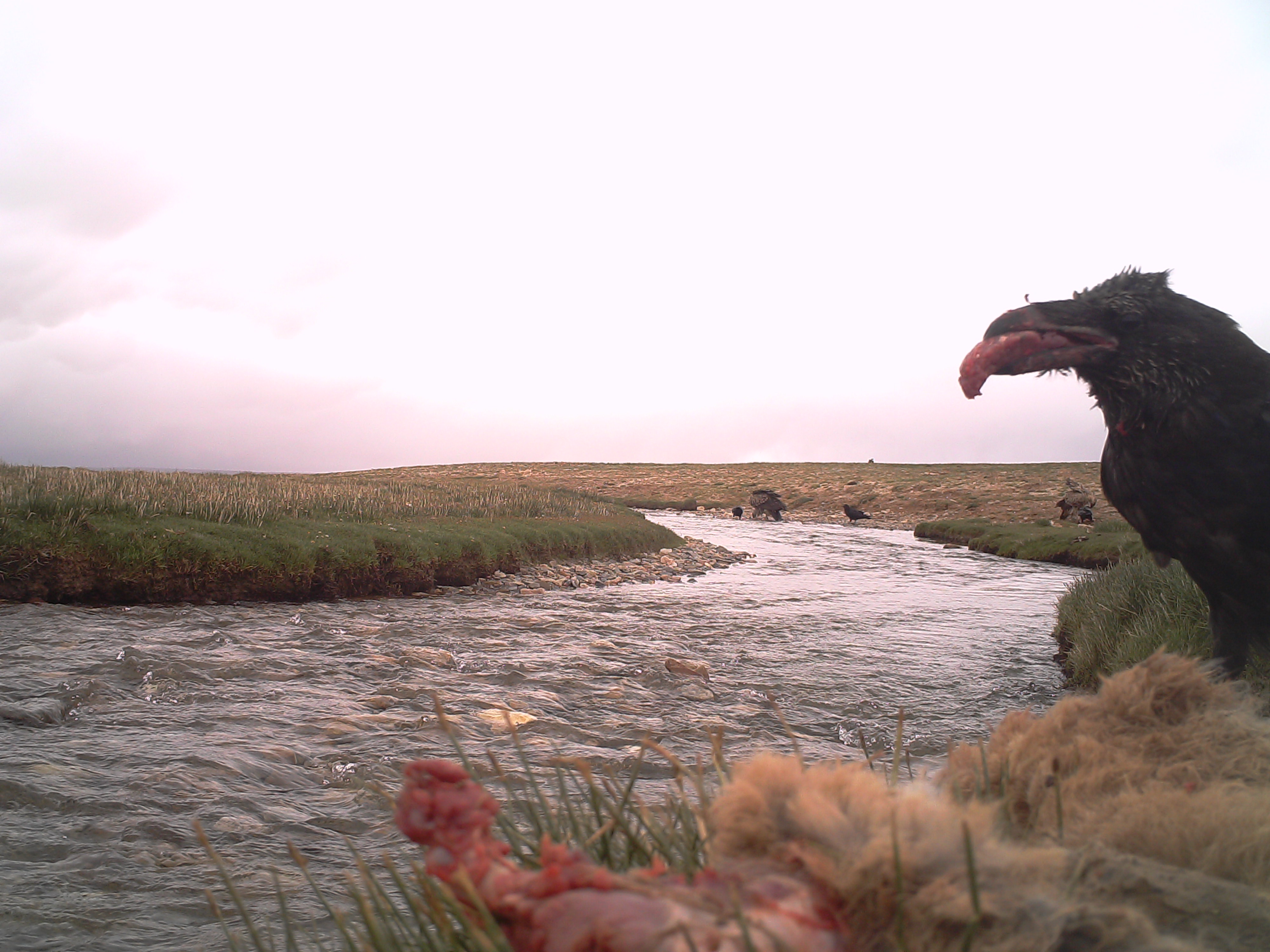

Supplement: Supplementary material 4 — Predators: Corvuscorax [file bdj-11-e107957-s004.jpg]
